# Editorial: Role of health economic data in policy making and reimbursement of new medical technologies, Volume II

**DOI:** 10.3389/fpubh.2023.1179300

**Published:** 2023-03-27

**Authors:** Mihajlo Jakovljevic, Tetsuji Yamada, Danko Grujic

**Affiliations:** ^1^Institute of Advanced Manufacturing Technologies, Peter the Great St. Petersburg Polytechnic University, St. Petersburg, Russia; ^2^Institute of Comparative Economic Studies, Hosei University, Tokyo, Japan; ^3^Department of Global Health Economics and Policy, University of Kragujevac, Kragujevac, Serbia; ^4^Rutgers, The State University of New Jersey, Camden, NJ, United States; ^5^Clinic for Cardiac Surgery, University Clinical Center of Serbia, Belgrade, Serbia

**Keywords:** Asia-Pacific, health economics, universal health coverage, health insurance, health expenditure, pharmaceutical expenditure, developing countries, ASEAN

Accelerated growth of health spending began in the 1960s exceeding the historical 4% GDP threshold. This phenomenon was noticed early on in mature market economies led by the US and during the following decades spread to many global regions. Health policymakers became increasingly exposed to new harsh challenges in the uneasy task to provide universal health coverage and decent equity of access to medical services. Among the most prominent demand-side issues are population aging, a rise of non-communicable diseases, and growing patient expectations. Supply-side causes include improvements in societal welfare and living standards, technological innovation in medicine, and continuing rapid urbanization in developing world regions. Successful insurance-based risk-sharing agreements made drug dispensing and medical service provision cheap or virtually free at the point of consumption in most OECD and many middle-income countries (1, 2). Also, the massive build-up of workforce capacities and strengthening of primary care and hospital networks contributed to the “supplier induced demand” phenomenon (2).

There is straightforward historical evidence of long-term growth in pharmaceutical and overall health spending both in absolute and GDP % terms worldwide (3). The accumulated constraints resulting from skyrocketing costs of care were felt in many areas of clinical medicine even among the richest societies (4, 5). Examples of expensive and hardly affordable novel therapeutic areas are orphan drugs indicated to treat rare diseases and targeted biologicals used in autoimmune disorders and cancer. Frequently denied access to even essential generic pharmaceuticals is still taking place, in particular in rural and suburban areas of low and middle-income countries (6, 7). These difficulties are worsened by the lack of evidence-based resource allocation strategies and less sustainable financing strategies (8).

The most comprehensive set of studies on the topic of challenging, complex, and less sustainable finance solutions for novel therapies are provided by Chinese universities. Xie et al. presented a survey of patients with metastatic urothelial carcinoma, where avelumab (a human anti-programmed cell death ligand 1 antibody against PD-L1) maintenance therapy (AVE) was a more cost-effective first-line treatment than the best supportive care (CON). In the entire population and the PD-L1-positive population, the incremental cost-effectiveness ratios of the AVE group were $38,369.50 and 16,150.29 per QALY, respectively. In the whole population and the PD-L1-positive population, the ICER of the AVE group was $241,610.25 and 100,528.29 per QALY, respectively. Decreasing the current avelumab prices by a significant percentage could maximize the cost-effectiveness of avelumab maintenance therapy.

Another similar research was conducted. Tislelizumab is a PD-1 (programmed cell death protein) inhibitor. Tislelizumab plus chemotherapy as a first-line treatment for advanced non-squamous non-small cell lung cancer (NSCLC) resulted in considerably longer life outcomes when compared to chemotherapy alone; nevertheless, information regarding its relative efficacy and the cost is inadequate. Tan et al. wanted to compare the cost-effectiveness of tislelizumab plus chemotherapy vs. chemotherapy alone in health care in China. Compared to chemotherapy alone, tislelizumab combined with chemotherapy improved QALYs by 0.64 and life-years by 1.48, with a cost increase of $16,631 per patient. The results were encouraging. In China, the combination of tislelizumab with chemotherapy is anticipated to be cost-effective as a first-line treatment for advanced non-squamous NSCLC.

China began implementing the consistency evaluation policy for generic pharmaceuticals in 2016. From a theoretical perspective, many experts anticipated that the legislation would encourage pharmaceutical businesses to expand R&D spending, but only some studies have been done. As a result, Wei et al. employed a difference-in-differences (DID) model and panel data from 111 A-share listed pharmaceutical firms from 2012 to 2020 to empirically investigate the influence of generic drug consistency evaluation policy on pharmaceutical firms' R&D investment intensity. The findings suggest that the legislation has a substantial favorable impact on enterprises' R&D investment intensity. Compared to prior academic research, this study presents empirical evidence for the consistency evaluation policy generic medications to encourage pharmaceutical enterprises to expand R&D investment and conducts an in-depth analysis of the policy's impact from the standpoint of heterogeneity.

Researchers from the Chinese University of Engineering Science worked on an intriguing topic involving the impact of payment methods on medical insurance expense control. The cost control effect of the dual difference (DID) model of medical insurance payment method was evaluated in that study, which used annual data from 27 provinces from 2013 to 2017. Failure to considerably lower the growth rate of medical insurance fund expenditure is not ideal for containing the excessive expansion of health insurance funds. As a result, enhancing control of medical expenses and improving control of medical insurance fund fees through payment method reform are effective strategies to strengthen the management of medical insurance funds.

A study of the cost-effectiveness of different immunization schedules with inactivated Sabin strain (sIPV) polio vaccine was conducted and analyzed to evaluate the cost-effectiveness of different sIPV immunization schedules, which could provide convincing evidence to change the poliovirus vaccine (PV) immunization strategies in China. Further research is needed into the long-term effectiveness and advantages of IPV-containing regimens in China (9).

Another intriguing study attempts to investigate whether the Chinese general public has a social preference for orphan pharmaceuticals and to quantify the personal quantitative trade-off between critical qualities of orphan drugs using a discrete choice experiment. A total of 323 people took part in this research. In conclusion of this research, in China, the general public does not regard rarity as a sufficient cause to merit special consideration in supporting orphan medications. While deciding on coverage, the public prioritized annual cost, disease severity, and medication side effects.

This Research Topic was created with a mission to tackle the core challenges for the provision of new medical technologies across the globe. The objective is to reveal some of the hidden underlying causes of unequal access to medicines as well as the growing proportion of out-of-pocket health spending in many world regions. In essence, the Topic belongs to the interdisciplinary sciences of pharmacoeconomics and health economics. Health policy considerations should be primarily focused on financing mechanisms and affordability of medicines and health care in general. Issues such as health insurance, reimbursement, and cost-containment strategies, and inequities in health care access may also be considered. Scientific contributions from all relevant stakeholders including academia, industry, and regulatory authorities are welcomed. This Topic has attracted a total of eight contributions among whom six were successfully published.

The editors hope these significant and diverse subject contributions will help expand existing knowledge. In addition, this is a unique opportunity to hold a public debate about Asian concerns regarding the future of health economics (10, 11). A heterogeneous group of authors from academia, the pharmaceutical, the medical device industries and governments aimed to present a comprehensive review of the advanced understanding of the Asian-Australasian economy (12, 13). We hope this collection of articles will pique the interest of aspiring Asian health economists and the general public.

## Author contributions

MJ has prepared the manuscript draft while MJ and TY have revised it for important intellectual content. All authors contributed to the article and approved the submitted version.
